# The influence of adiposity status and the trajectories of obesity indicators on earlier puberty onset in girls: a longitudinal cohort study in Shanghai, China

**DOI:** 10.1186/s12889-025-25122-9

**Published:** 2025-11-27

**Authors:** Chen Liu, Tianfeng Wu, Xiaowei Liu, Cun Qin, Yao Xu, Pinqing Bai, Yaping Ren

**Affiliations:** 1 Department of School Health, Food Nutrition and Safety, Shanghai Pudong New Area Center for Disease Control and Prevention (Shanghai Pudong New Area Health Supervision Institute), Zhangyang Road 3039, Shanghai, 200136 China; 2 Administrative Office, Shanghai Pudong New Area Center for Disease Control and Prevention (Shanghai Pudong New Area Health Supervision Institute), Zhangyang Road 3039, Shanghai, 200136 China

**Keywords:** Obesity status, Overweight and obesity, Trajectory, Early puberty onset

## Abstract

**Background:**

A global trend toward earlier onset of puberty in girls has been reported, with increasing prevalence observed in countries such as Denmark, South Korea, and China. Early puberty is associated with a range of adverse physical and psychological outcomes. Obesity is a key modifiable factor potentially contributing to early pubertal development; however, most existing evidence is based on cross-sectional studies. In China, longitudinal studies using objective measurements are scarce, particularly those examining the impact of obesity status across multiple time points. This study aimed to investigate the associations between prepubertal obesity, trajectories of obesity indicators, and early pubertal development in girls using data from a school-based cohort in Shanghai.

**Methods:**

A total of 1, 030 first-grade girls in Pudong, Shanghai were enrolled in 2020 and followed annually to assess body mass index (BMI), waist circumference (WC), breast, and pubic hair development. Group-based trajectory modeling identified distinct body mass index and waist circumference trajectories before puberty, and log-binomial regression was used to evaluate their associations with early puberty.

**Results:**

Girls who were overweight or obese before puberty had a higher risk of early pubic hair development compared to non-overweight peers, while only obesity was associated with earlier breast development. Prepubertal abdominal obesity was also linked to increased risks of early breast and pubic hair development. BMI and WC trajectories were categorized into gradual, persistent, and rapid increase groups. Compared to the gradual group, girls with persistent and rapid increases in BMI (RR: 2.28, 995%CI: 1.40–3.72; RR: 3.34, 95%CI: 1.79–6.23) and WC (RR: 2.43, 95%CI: 1.52–3.89; RR: 2.68, 95%CI: 1.22–5.88) had significantly higher risks of early pubic hair development; persistent BMI (RR: 1.70, 95%CI: 1.25–2.29) and WC (RR: 1.81, 95%CI: 1.34–2.44) increases were also associated with earlier breast development, while rapid BMI/WC increases did not accelerate breast development compared to gradual trajectories.

**Conclusion:**

Sustained gains in BMI or WC, rather than single-point obesity, substantially heighten the risk of earlier pubertal onset in girls, underscoring the value of longitudinal weight monitoring for early intervention.

## Background

A long-term trend of early puberty in girls was observed in many countries [1, 2]. Although differences in the prevalence of early puberty across countries may be related to variations in racial and social backgrounds, global studies consistently indicate a trend toward earlier onset of puberty in children. A nationwide registry-based study in Denmark showed that from 1998 to 2017, the incidence of early puberty in girls increased from 2.6 per 100,000 to 14.6 per 100,000 [3]. In South Korea, surveys revealed that the incidence of central precocious puberty (CPP) in girls rose from 0.33 per 100,000 in 2004 to 5.04 per 100,000 in 2010 [4], with an incidence of 26.28 per 100,000 and a prevalence of 41.06 per 100,000 reported for girls from 2008 to 2014 [5]. A survey on early puberty in Taiwan, China, indicated that the crude prevalence in girls increased from 13.56 per 100,000 in 2000 to 110.95 per 100,000 in 2013 [6]. A 2021 school-based study in China, reported a early puberty rate of 13.43% in girls, with rates of 11.30% among urban girls and 14.16% among suburban girls [7]. In addition, recent evidence has provided updated estimates of pubertal onset in Chinese girls. A recent large-scale national study reported that the median age of Tanner stage II breast development was 9.65 years [8]. Consistent with this, a systematic review estimated a pooled median age of 9.6 years across Chinese regions and documented a secular trend toward earlier onset [9]. These findings highlight the importance of focusing on the early school years as a critical developmental window for capturing the initiation of puberty in Chinese girls.

Physiologically, children with early puberty experience accelerated growth in height, weight, and bone age, which may result in premature epiphyseal closure and a shortened growth window, potentially compromising adult height and, in extreme cases, causing dwarfism. Early puberty has also been associated with increased long-term risks of hormone-related cancers, including breast and uterine cancers [10, 11]. Furthermore, from a sociopsychological perspective, the psychological changes caused by early puberty can impose a significant psychological burden on children, potentially leading to early pubertal behaviors or juvenile pubertal offenses, among other social issues [12]. Given the plasticity of pubertal timing, identifying modifiable factors influencing early puberty is crucial for informing preventive strategies.

The timing of puberty in girls is influenced by a combination of genetic, environmental, familial, and social factors, but the underlying mechanisms remain only partially understood [13]. In particular, the concurrent trends of declining age at early puberty and increasing prevalence of obesity have generated interest in the association between childhood obesity and early puberty in girls [14]. Obesity may influence pubertal development through hormonal changes, metabolic effects of adipose tissue, and alterations in growth factor regulation [15, 16]. Most studies in developed countries support a close relationship between the rate of pubertal maturation and the degree of obesity in girls [1, 17]. The pubertal development of girls can be characterized by multiple features—breast development, pubic and axillary hair growth, menarche, and the maturation of internal reproductive organs. However, the association between different levels of obesity and specific pubertal developmental features remains inconclusive. Evidence from most cross-sectional studies suggests that girls with obesity are more likely to exhibit earlier breast and pubic hair development, potentially due to increased obesity [18, 19]. In contrast, a cohort study in the United Kingdom reported that obesity was linked to earlier pubic hair development but not breast development, suggesting that different pubertal markers may be influenced through distinct biological pathways [20].

In China, evidence regarding the relationship between childhood obesity and pubertal timing is still limited. Most available studies are cross-sectional in design and rely on single-point assessments of obesity status. These measures are susceptible to short-term fluctuations and may not adequately reflect dynamic growth patterns. By contrast, longitudinal trajectories of obesity provide a more comprehensive picture of cumulative exposure and weight gain velocity, offering greater insight into identifying high-risk growth patterns. Additionally, cohort studies with adequately large sample sizes are relatively scarce. Furthermore, many studies rely on questionnaire-based assessments to determine the initiation of pubertal development, lacking confirmation through gold-standard methods such as physical examinations, which raises concerns about the accuracy of these findings. Based on a cohort design, this study utilized anthropometric characteristics, including waist circumference, body mass index, and their changing trajectories, to estimate the impact of obesity on early pubertal onset in girls. It aims to move beyond assessing obesity at a single time point, providing new evidence on how changes in obesity status influence early pubertal onset in girls, and offering a theoretical basis for early interventions to prevent early puberty.

## Methods

### Subjects

This study was part of a prospective cohort initiated in September 2020, targeting first-grade students from 13 primary schools across different regions (urban, urban–rural, rural) in Pudong District, Shanghai. The study aimed to monitor pubertal development and identify influencing factors. A total of 1,232 girls were invited, and 1,030 completed all three follow-up assessments by December 2023, with 202 participants lost due to school transfer or withdrawal. Annual physical examinations were conducted over four school years from 2020 to 2023—one prepubertal baseline in first grade and three annual follow-ups (grades 2–4), forming a four-school-year observation window. This observation window was deliberately chosen to establish a true prepubertal baseline and to span the typical age of pubertal onset among Chinese girls, thereby allowing the identification of early-onset cases while maintaining temporality between prepubertal adiposity and subsequent puberty initiation. Pubertal development assessments began when the girls entered third grade and were conducted twice. Written informed consent was obtained from all participants and their guardians. Consent materials were distributed via school health teachers, and guardians were encouraged to discuss participation with their children. Participation was voluntary and confidentiality was strictly maintained.

### Survey instruments and variables

The study used a self-developed questionnaire to assess girls’sociodemographic and birth-related variables: age, birth weight, region of residence, maternal parity, gestational weight gain, only-child status, parental education (junior high or below, senior high or equivalent, bachelor’s degree or above), and household monthly income(see Supplementary File 1 for the English version).

Height and weight were measured using a calibrated electronic stadiometer with 0.1 cm and 0.1 kg precision, respectively. Girls were measured twice by trained examiners without shoes and in light clothing; the average of the two readings was used. Waist circumference (WC) was measured using a non-elastic tape at the midpoint between the lower rib and the iliac crest after normal expiration. Measurements were taken twice; if the difference exceeded 0.5 cm, a third reading was taken, and the closest two were averaged. Body mass index (BMI) was calculated as weight in kilograms divided by height in meters squared (kg/m²). Overweight and obesity were defined according to the 2018 Chinese Screening Guidelines for School-Age Children and Adolescents [21]. Abdominal obesity was defined as WC ≥ age-and sex-specific 90th percentile (P90), based on national reference data for Chinese children [22].

Pubertal development was assessed by two independently trained physicians according to Tanner staging [1]. Breast development was assessed through inspection and palpation to differentiate between glandular and adipose tissue, using Tanner charts as a reference. Two examiners assessed each girl simultaneously; in cases of disagreement, a re-examination was conducted until consensus was reached. The onset of puberty was defined as the age at which a girl reached Tanner stage II for breast development, recognized as the initial sign of puberty [23]. Early puberty was defined as onset age falling in the first quartile of the age distribution within the cohort [24]. The pubertal development outcomes in this study were based on the physical examination results of fourth-grade students.

### Quality control

Standardized electronic questionnaires were distributed through a student health management platform, which enforced logic checks and mandatory fields to prevent missing or invalid responses. A trained medical team conducted physical exams using uniform instruments and protocols. All exams were performed by same-sex clinicians. Data were double-entered and reviewed for consistency; abnormal values were verified against original records.

### Statistical analysis

Descriptive statistics were presented as means and SD for continuous variables and as numbers and percentages for categorical variables. Differences between different groups were assessed using t test and chi-square test. The log-binomial regression was applied to report risk ratios (RR) and 95% confidence intervals (CI) to analyze the relationship between prepubertal anthropometric profiles, their trajectories and risk of earlier puberty onset. For point-in-time overweight and obesity rates, a single physical examination conducted prior to the calculated onset age of each aspect of pubertal development should be selected.

Based on SAS9.4, a group-based trajectory model (GBTM) was used to model BMI and WC in girls before the onset of puberty and to fit the best trajectory model [25]. Model selection was guided by the Bayesian Information Criterion (BIC), average posterior probabilities (≥ 0.7), and reasonable group sizes [26, 27]. Models with one to four groups and polynomial terms (linear, quadratic, cubic) were tested. The optimal model contained three trajectory groups for both BMI and WC, reflecting gradual, persistent, and rapid increases. These trajectories captured distinct patterns of obesity change, illustrating the heterogeneous nature of BMI and WC development.

All statistical tests were two-sided, and *P* < 0.05 was regarded as significant.

### Ethics approval

This study was approved by the Pudong New Area CDC/School of Public Health Fudan University Ethics Committee (2022-TYSQ-03–151).

## Results

### Comparison of Characteristics and Baseline Physical Examination of Enrolled and Dropped-out Girls

A total of 1030 girls completed the 3-year follow-up and were eventually included in the analysis. The mean age was 6.46 ± 0.37 years for girls at baseline. The mean of BMI, waist circumference was 15.91 ± 2.17 and 51.74 ± 5.59, respectively. The analysis showed that there were statistically significant differences in region and number of deliveries between enrolled and dropped-out girls (*χ*^*2*^ = 17.01, *P* < 0.001; *χ*^*2*^ = 4.23, *P* < 0.05). No statistically significant differences were found in other characteristics (*P* > 0.05) (Table 1).

**Table 1:** Comparison of characteristics and baseline physical examination of enrolled and dropped-out girls

Characteristics	Enrolled (*n* = 1030)	Dropped-out (*n* = 202)	t/χ^2^
Age(years)	6.46 ± 0.37	6.49 ± 0.41	−0.51
Height(cm)	121.86 ± 5.36	122.66 ± 5.54	−0.98
Weight(kg)	23.79 ± 4.51	24.28 ± 5.86	−0.70
Body mass index (kg/m^2^)	15.91 ± 2.17	16.15 ± 3.58	−0.42
Waist circumference(cm)	51.74 ± 5.59	53.20 ± 7.56	−1.70
Birth weight(kg)	3.29 ± 0.47	3.33 ± 0.49	−1.24
Gestational weight gain (kg)	14.70 ± 4.72	14.85 ± 5.15	−0.41
Region			
Urban area	350(33.98%)	95(47.03%)	17.01***
Urban-rural area	527(51.17%)	72(35.64%)	
Rural area	153(14.85%)	35(17.33%)	
Maternal parity			
First time	803(77.96%)	144(71.29%)	4.23*
Non-first time	227(22.04%)	58(28.71%)	
Only child			
Yes	499(48.45%)	101(50.00%)	0.16
No	531(51.55%)	101(50.00%)	
Average monthly household(RMB)			
< 4000	179(17.38%)	36(17.82%)	3.18
4000–9999	473(45.92%)	81(40.10%)	
≥ 10,000	378(36.70%)	85(42.08%)	
Paternal education level			
Junior high school degree or below	143(13.88%)	28(13.86%)	0.46
Senior high school or equivalent	429(41.65%)	89(44.06%)	
Bachelor’s degree or above	458(44.47%)	85(42.08%)	
Maternal education level			
Junior high school degree or below	168(16.31%)	44(21.78%)	3.74
Senior high school or equivalent	444(43.11%)	82(40.59%)	
Bachelor’s degree or above	418(40.58%)	76(37.63%)	

*** *P* < 0.001, **P < 0.01, *P < 0.05

### Trajectory patterns of BMI and waist circumference prior to puberty

**Fig. 1:**
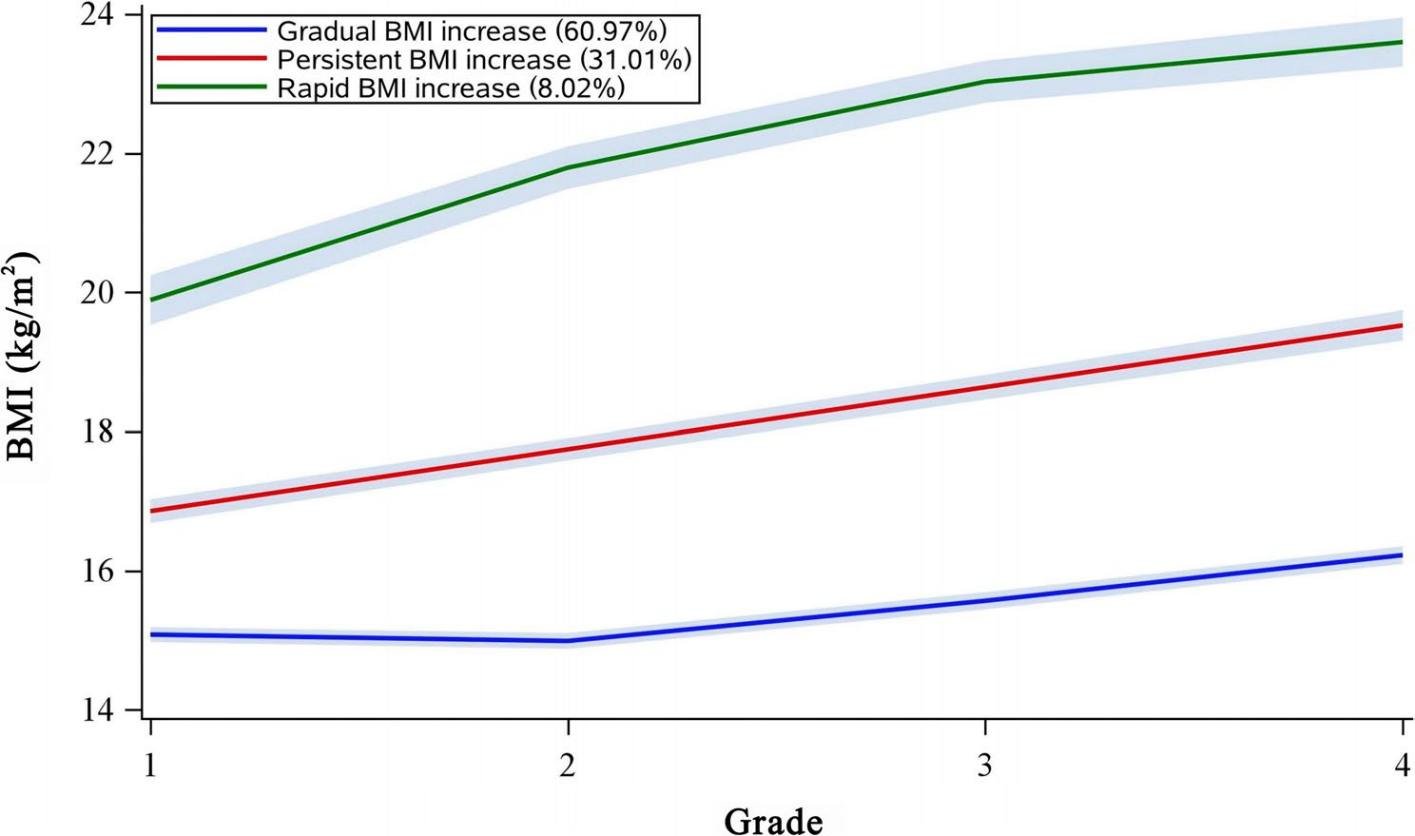
The trajectories of BMI from baseline to the end of follow-up

**Fig. 2:**
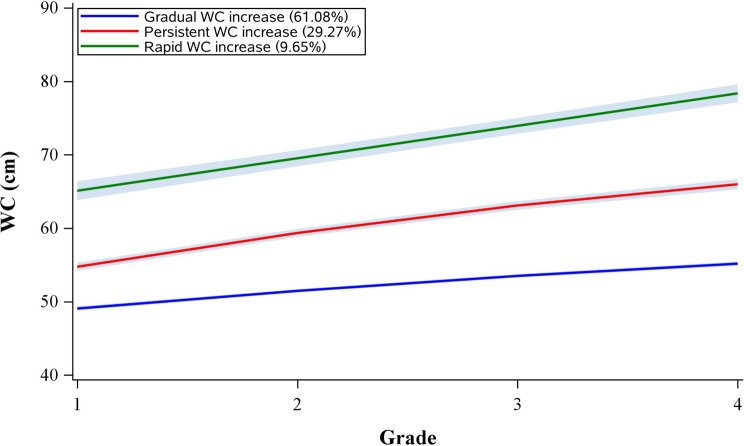
The trajectories of WC from baseline to the end of follow-up

Based on the trajectory model analysis, the variation trends of each BMI and WC trajectory subgroup are presented in Figs. 1 and 2. Girls were categorized into three BMI trajectory groups: [1] a gradual BMI increase group, which started at a low level and exhibited a relatively slow increase over time; [2] a persistent BMI increase group, which started at a moderate level and demonstrated a stable increase; and [3] a rapid BMI increase group, which began at a high level and showed a rapid upward trend.

Similarly, three WC trajectory groups were identified: gradual WC increase, persistent WC increase, and rapid WC increase.

In the BMI trajectory model, the proportions of girls in the gradual, persistent, and rapid BMI increase groups were 60.97%, 31.01%, and 8.02%, respectively, with corresponding BMI ranges of 14.99–16.22, 16.86–19.42, and 19.90–23.58 kg/m² (Fig. 1). In the WC trajectory model, the proportions were 61.08% for the gradual WC increase group, 29.27% for the persistent group, and 9.65% for the rapid group, with WC ranges of 49.11–55.11, 54.94–65.78, and 65.16–77.76 cm, respectively (Fig. 2).

### Prepubertal obesity Status, trajectories and early puberty development

There were differences in the incidence of early puberty development among different trajectory groups. The incidence of breast and pubic hair development were significantly higher in relatively high-increasing trajectory group of BMI (*χ*^*2*^ = 13.20, *P* = 0.001; *χ*^*2*^ = 20.09, *P* < 0.001), WC (*χ*^*2*^ = 17.75, *P* < 0.001; *χ*^*2*^ = 20.87, *P* < 0.001), than that in gradual BMI increase group (Table 2).

**Table 2:** The incidence of early puberty development in different trajectory groups of anthropometric profiles

Anthropometric profiles	Trajectory groups	Premature breast development		Premature pubic hair development	
		Incidence	*χ2*	Incidence	*χ2*
Body mass index	Gradual BMI increase	15.53%	13.20**	5.87%	20.09***
	Persistent BMI increase	26.07%		12.82%	
	Rapid BMI increase	25.00%		19.44%	
Waist circumference	Gradual WC increase	15.30%	17.75***	5.87%	20.87***
	Persistent WC increase	27.75%		14.98%	
	Rapid WC increase	26.67%		17.78%	

*** *P* < 0.001, **P < 0.01, *P < 0.05

In analyses stratified by prepubertal obesity status, overweight girls had a higher risk of early pubic hair development compared to their non-overweight peers (RR: 2.32, 95%CI: 1.40–3.73), while no significant difference was observed for breast development. Obese girls were at increased risk of both premature breast and pubic hair development relative to non-overweight girls (RR: 1.83, 95%CI: 1.30–2.56; RR: 3.32, 95%CI: 1.77–6.24). Similarly, girls with abdominal obesity were more likely to experience earlier onset of breast and pubic hair development compared to those without abdominal obesity (RR: 1.61, 95%CI: 1.20–2.15; RR: 1.91; 95%, CI: 1.22–3.00.22.00) (Table 3).

**Table 3:** Prepubertal obesity status and growth trajectories in relation to early pubertal onset

Anthropometric profiles	Incidence of premature breast development		Incidence of premature pubic hair development	
	Model 1	Model 2	Model 1	Model 2
Obesity status in prepuberty				
Body mass index				
Non-overweight	1 (reference)	1 (reference)	1 (reference)	1 (reference)
Overweight	1.43(0.96,2.12)	1.43(0.96,2.13)	2.18(1.35,3.52)	2.32(1.40,3.73)
Obesity	1.92(1.40,2.63)	1.83(1.30,2.56)	3.31(1.85,5.92)	3.32(1.77,6.24)
Waist circumference				
Non–abdominal obesity	1 (reference)	1 (reference)	1 (reference)	1 (reference)
Abdominal obesity	1.66(1.26,2.18)	1.61(1.20,2.15)	2.06(1.34,3.17)	1.91(1.22,3.00)
Trajectory groups				
Body mass index				
Gradual BMI increase	1 (reference)	1 (reference)	1 (reference)	1 (reference)
Persistent BMI increase	1.68(1.25,2.25)	1.70(1.25,2.29)	2.18(1.35,3.52)	2.28(1.40,3.72)
Rapid BMI increase	1.61(1.03,2.52)	1.47(0.91,2.37)	3.31(1.85,5.92)	3.34(1.79,6.23)
Waist circumference				
Gradual WC increase	1 (reference)	1 (reference)	1 (reference)	1 (reference)
Persistent WC increase	1.81(1.36,2.41)	1.81(1.34,2.44)	2.55(1.62,4.01)	2.43(1.52,3.89)
Rapid WC increase	1.74(1.03,2.94)	1.52(0.87,2.66)	3.03(1.49,6.16)	2.68(1.22,5.88)

Table shows the risk ratios and 95%CI. Model 1 was the crude model. Model 2 was adjusted for living region, maternal parity, maternal gestational weight gain, only child, birth weight, average monthly household, and parents’ education level

Girls with a persistent increase trajectory of BMI were at a higher risk of premature breast development (RR: 1.70, 95%CI: 1.25–2.29). Similarly, those with persistent or rapid increase trajectories of BMI were more likely to experience premature pubic hair development (RR: 2.28, 95%CI: 1.40–3.72; RR: 3.34, 95%CI: 1.79–6.23). Likewise, girls with a persistent increase trajectory of WC were at higher risk of premature breast development (RR: 1.81, 95%CI: 1.34–2.44), while those with persistent or rapid increases in WC showed elevated risks of premature pubic hair development (RR: 2.43, 95%CI: 1.52–3.89; RR: 2.68, 95%CI: 1.22–5.88) (Table 3).

## Discussion

This study dynamically and continuously tracked two obesity indicators in girls and evaluated how their distinct trajectories relate to two pubertal maturation outcomes. By moving beyond single, pre-pubertal snapshots, this approach better captures high-risk growth patterns and population heterogeneity. The results demonstrate that prepubertal obesity in girls increases the risk of early pubertal development during puberty. In addition, persistent generalized obesity before puberty and persistent abdominal obesity further increase the risk of early breast development in girls. Children with higher growth trajectories in obesity status face a significantly increased risk of premature pubic hair development.

For point-in-time obesity before adolescence, both general obesity and abdominal obesity increase the risk of early breast and pubic hair development in girls, which is consistent with most studies on the association between obesity and earlier puberty onset in girls [17, 18]. However, this study found no significant difference in the risk of early breast development between overweight girls and normal-weight girls at prepubertal time points, which differs from some previous research findings [17, 28]. This may suggest that a single point-in-time measure is insufficiently sensitive in capturing the impact of overweight on early breast development in girls. Additionally, in China, most studies on the relationship between obesity and early puberty in girls are cross-sectional [17, 29]. Given the complex relationship between obesity and puberty development, such studies cannot establish causality, highlighting a limitation in this area of research.

This study explored the trajectories of continuous changes in multiple obesity indicators in girls and their association with breast and pubic hair development, yielding notable findings. Compared to girls with stable BMI and waist circumference, those with persistently increasing BMI and waist circumference exhibited a higher risk of early breast and pubic hair development. A study from Taiwan also reported that girls with prolonged overweight/obesity had the highest risk of early puberty, which aligns with the findings of this study [30]. Among girls with initially high levels of BMI and waist circumference who maintained a rapid growth trend, the risk of early pubic hair development was the highest, however, this pattern was not observed in breast development. Fewer previous studies abroad on obesity trajectories and early adolescence have reached similar conclusions [31]. This discrepancy may be attributed to the influence of various internal and external factors, such as ethnicity, socioeconomic status, and other regional differences, which contribute to the variability in findings across different populations.

Previous studies have shown that although both breast and pubic hair development mark the onset of sexual maturation in girls, they are regulated by distinct physiological axes and hormones and remain relatively independent during the pubertal process [17, 32]. Breast development is primarily regulated by the hypothalamic-pituitary-gonadal (HPG) axis, with estrogen serving as the key driver. In contrast, pubic hair development is regulated by the hypothalamic-pituitary-adrenal (HPA) axis and is closely associated with androgens. In obese girls, increased adipose tissue leads to elevated aromatase activity, resulting in higher estrogen levels that accelerate breast development. Obesity is also strongly linked to insulin resistance, which stimulates excessive androgen secretion by the ovaries, thereby promoting pubic hair development. Additionally, leptin, a hormone secreted by adipose tissue, plays a crucial role in sexual maturation. Additionally, leptin secreted by adipose tissue and elevated levels of insulin-like growth factor-1 (IGF-1) in obese girls can also accelerate the development of both breasts and pubic hair. These differences in regulatory mechanisms result in varying sensitivities to obesity, which may partially explain the asynchrony observed in the impact of obesity on breast and pubic hair development in this study.

Studies have shown that excessive obesity in boys can affect sexual development, whereas similar findings are less frequently observed in girls [33, 34]. In the present study, we found that girls with initially higher obesity levels and those experiencing rapid increases in obesity did not exhibit a significantly higher rate of early breast development compared to girls with more gradual weight trajectories. One possible explanation is that excessively high leptin levels may lead to leptin resistance, thereby attenuating its effect on the hypothalamus. Additionally, obesity-induced inflammation may not necessarily accelerate the process of breast development [35].

Additionally, the choice of obesity indicators can influence the exploration of the relationship between obesity and early puberty. Some previous studies have focused solely on BMI, overlooking other indicators such as waist circumference, which reflect different types of obesity and fat distribution. Particularly for children, waist circumference, as an easily measurable parameter, is more effective in identifying obese children and assessing fat distribution [36]. Studies have indicated that excessive fat accumulation in the abdominal or central region increases the risk of metabolic syndromes, such as dyslipidemia and insulin resistance, thereby influencing pubertal development in girls [37].

Nutritional factors, genetic predisposition, and endocrine-disrupting environmental substances are potential mediators linking obesity to sexual development. Therefore, future research should aim to explore the bidirectional relationship and underlying mechanisms between obesity and the initiation of sexual development in children and adolescents [38].

The long-term trends of overweight and obesity during childhood influence the onset of early puberty, emphasizing the necessity of sustained overweight and obesity management in children. Future efforts should focus on increasing the frequency of monitoring multidimensional obesity indicators to facilitate the early identification of high-risk children based on surveillance data, ensuring timely intervention during the prepubertal window. Additionally, healthcare institutions should collaborate with schools, families, and society at multiple levels to implement comprehensive weight intervention strategies throughout the entire growth and developmental period. Strengthening such interventions helps regulate the timing and pace of pubertal development in children and adolescents, ultimately promoting their overall physical and mental well-being.

### Strengths and limitations

This study has several strengths. Its prospective, longitudinal cohort covered four school years, which established a true prepubertal baseline and ensured temporality between adiposity and the onset of puberty. Repeated objective anthropometric measurements (BMI and waist circumference) obtained through standardized, school-based examinations enabled trajectory modeling to characterize cumulative exposure and growth velocity, providing a more comprehensive understanding than single-time assessments. Pubertal development was assessed by trained physicians using visual inspection and palpation with dual-examiner agreement, enhancing measurement reliability.

However, some limitations should be acknowledged. First, pubertal assessments were conducted annually starting in third grade, which may have missed some transitions. Second, the observation window ends at grade 4 (9–10 years), which may underestimate later-onset events. Follow-up will continue into later adolescence to capture additional milestones. Lastly, the study sample was drawn from Pudong, Shanghai, limiting generalizability to rural areas or other regions with different lifestyle and environmental exposures.

## Conclusion

The prospective cohort study design allowed us to unravel the potential impact of prepubertal anthropometric characteristics on early puberty. Overweight and obesity, and abdominal obesity were associated with earlier puberty in girls. In addition, children with persistent growth trajectories in anthropometric characteristics were more likely to be at risk for early puberty. Interventions aimed at reducing childhood obesity levels and focusing on long-term healthy obesity status may help to prevent early puberty, adult obesity, and related adverse health outcomes.

## Data Availability

The datasets generated and/or analyzed during the current study are not publicly available due to participant privacy and confidentiality concerns, but are available from the corresponding author on reasonable request.

## Abbreviations

BMI: Body Mass Index
WC: Waist Circumference
GBTM: Group-based Trajectory Model
RR: Relative Risk
CI: Confidence Interval
SD: Standard Deviation

## References

[1] Eckert-Lind C, Busch AS, Petersen JH, Biro FM, Butler G, Bräuner EV, et al. Worldwide secular trends in age at pubertal onset assessed by breast development among girls: a systematic review and meta-analysis. JAMA Pediatr. 2020;174(4):e195881. 32040143 10.1001/jamapediatrics.2019.5881 PMC7042934

[2] Brix N, Ernst A, Lauridsen LLB, Parner E, Støvring H, Olsen J, et al. Timing of puberty in boys and girls: a population-based study. Paediatr Perinat Epidemiol. 2019;33(1):70–8. 30307620 10.1111/ppe.12507 PMC6378593

[3] Bräuner EV, Busch AS, Eckert-Lind C, Koch T, Hickey M, Juul A. Trends in the incidence of central precocious puberty and normal variant puberty among children in Denmark, 1998 to 2017. JAMA Netw Open. 2020;3(10):e2015665. 33044548 10.1001/jamanetworkopen.2020.15665 PMC7550972

[4] Kim SH, Huh K, Won S, Lee KW, Park MJ. A significant increase in the incidence of central precocious puberty among Korean girls from 2004 to 2010. PLoS ONE. 2015;10(11):e0141844. 26539988 10.1371/journal.pone.0141844 PMC4634943

[5] Kim YJ, Kwon A, Jung MK, Kim KE, Suh J, Chae HW, et al. Incidence and prevalence of central precocious puberty in Korea: an epidemiologic study based on a National database. J Pediatr. 2019;208:221–8. 30857777 10.1016/j.jpeds.2018.12.022

[6] Su PH, Huang JY, Li CS, Chang HP. The age distribution among children seeking medical treatment for precocious puberty in Taiwan. Int J Environ Res Public Health. 2020;17(18):6765. 32957428 10.3390/ijerph17186765 PMC7559721

[7] Liu Y, Yu T, Li X, Pan D, Lai X, Chen Y, et al. Prevalence of precocious puberty among Chinese children: a school population-based study. Endocrine. 2021;72(2):573–81. 33528762 10.1007/s12020-021-02630-3

[8] Liang X, Huang K, Dong G, Chen R, Chen S, Zheng R et al. Current Pubertal Development in Chinese Children and the Impact of Overnutrition, Lifestyle, and Perinatal Factors. J Clin Endocrinol Metab. 2023;108(9):2282–9. 10.1210/clinem/dgad102 36881937

[9] Shu W, Zong X, Li H. Secular trends in age at pubertal onset assessed by breast development among Chinese girls: A systematic review. Front Endocrinol (Lausanne). 2022;13:1042122. 36506059 10.3389/fendo.2022.1042122 PMC9729541

[10] Lee S, Kim Y, Han M. Influence of waist circumference measurement site on visceral fat and metabolic risk in youth. J Obes Metab Syndr. 2022;31(4):296–302. 36274244 10.7570/jomes22046 PMC9828705

[11] Matłosz P, Wyszyńska J, Asif M, Szybisty A, Aslam M, Mazur A, et al. Prevalence of overweight, obesity, abdominal obesity, and obesity-related risk factors in Polish preschool children: a cross-sectional study. J Clin Med. 2021;10(4):790. 33669323 10.3390/jcm10040790 PMC7920301

[12] Andrews RR, Anderson KR, Fry JL. Sex-specific variation in metabolic responses to diet. Nutrients. 2024;16(17):2921. 39275236 10.3390/nu16172921 PMC11397081

[13] Spaziani M, Tarantino C, Tahani N, Gianfrilli D, Sbardella E, Lenzi A, et al. Hypothalamo-pituitary axis and puberty. Mol Cell Endocrinol. 2021;520:111094. 33271219 10.1016/j.mce.2020.111094

[14] Jebeile H, Kelly AS, O’Malley G, Baur LA. Obesity in children and adolescents: epidemiology, causes, assessment, and management. Lancet Diabetes Endocrinol. 2022;10(5):351–65. 35248172 10.1016/S2213-8587(22)00047-X PMC9831747

[15] Chung ST, Krenek A, Magge SN. Childhood obesity and cardiovascular disease risk. Curr Atheroscler Rep. 2023;25(7):405–15. 10.1007/s11883-023-01111-4 PMC10230147 37256483

[16] Sluijs EMF van, Ekelund U, Crochemore-Silva I, Guthold R, Ha A, Lubans D, et al. Physical activity behaviours in adolescence: current evidence and opportunities for intervention. The Lancet. 2021398(10298):429–42. 10.1016/S0140-6736(21)01259-9 PMC7612669 34302767

[17] Zhou X, Hu Y, Yang Z, Gong Z, Zhang S, Liu X, et al. Overweight/obesity in childhood and the risk of early puberty: a systematic review and meta-analysis. Front Pediatr. 2022;10:795596. 35722495 10.3389/fped.2022.795596 PMC9203728

[18] Zhang Y, Yuan X, Yang X, Lin X, Cai C, Chen S, et al. Associations of obesity with growth and puberty in children: a cross-sectional study in Fuzhou, China. Int J Public Health. 2023;68:1605433. 37255545 10.3389/ijph.2023.1605433 PMC10225596

[19] Aghaee S, Deardorff J, Quesenberry CP, Greenspan LC, Kushi LH, Kubo A. Associations between childhood obesity and pubertal timing stratified by sex and race/ethnicity. Am J Epidemiol. 2022;191(12):2026–36. 35998084 10.1093/aje/kwac148 PMC10144668

[20] O’Keeffe LM, Frysz M, Bell JA, Howe LD, Fraser A. Puberty timing and adiposity change across childhood and adolescence: disentangling cause and consequence. Hum Reprod. 2020;35(12):2784–92. 33242326 10.1093/humrep/deaa213 PMC7744159

[21] National Health and Family Planning Commission of the. People’s Republic of China. WS/T 586–2018 Screening for Overweight and Obesity Among School-Age Children and Adolescents. 2018.

[22] Zhao D, Zhou J, Su D, Li Y, Sun W, Tan B, et al. Combined associations of general obesity and central obesity with hypertension stages and phenotypes among children and adolescents in Zhejiang, China. J Clin Hypertens. 2023;25(11):983–92. 10.1111/jch.14733 PMC10631097 37787088

[23] Tanner JM, Whitehouse RH. Clinical longitudinal standards for height, weight, height velocity, weight velocity, and stages of puberty. Arch Dis Child. 1976;51(3):170–9. 952550 10.1136/adc.51.3.170 PMC1545912

[24] Li Y, Gao D, Chen M, Ma Y, Chen L, Ma J, et al. Association between healthy lifestyle pattern and early onset of puberty: based on a longitudinal follow-up study. Br J Nutr. 2022;128(12):2320–9. 35236516 10.1017/S0007114522000563

[25] Andruff H, Carraro N, Thompson A, Gaudreau P, Louvet B. Latent class growth modelling: a tutorial. TQMP. 2009;5(1):11–24.

[26] Mésidor M, Rousseau MC, O’Loughlin J, Sylvestre MP. Does group-based trajectory modeling estimate spurious trajectories? BMC Med Res Methodol. 2022;14(1):194. 10.1186/s12874-022-01622-9 PMC9281109 35836129

[27] Li QQ, Huang J, Cai D, Chou WC, Zeeshan M, Chu C, et al. Prenatal exposure to legacy and alternative per- and polyfluoroalkyl substances and neuropsychological development trajectories over the first 3 years of life. Environ Sci Technol. 2023;57(9):3746–57. 36800558 10.1021/acs.est.2c07807

[28] Fang J, Yuan J, Zhang D, Liu W, Su P, Wan Y, et al. Casual associations and shape between prepuberty body mass index and early onset of puberty: a Mendelian randomization and dose-response relationship analysis. Front Endocrinol (Lausanne). 2022;13:853494. 35360058 10.3389/fendo.2022.853494 PMC8964141

[29] Huiling LYU, Xi W, Jiale HU, Di H, a. N, Jia HU, Hui S. Association between different types of obesity and puberty timing in primary and secondary school students in Suzhou City. Zgxxws. 2023;44(12):1848–52.

[30] Fan HY, Lee YL, Hsieh RH, Yang C, Chen YC. Body mass index growth trajectories, early pubertal maturation, and short stature. Pediatr Res. 2020;88(1):117–24. 10.1038/s41390-019-0690-3 31791040

[31] Kim MR, Jung MK, Yoo EG. Slower progression of central puberty in overweight girls presenting with precocious breast development. Ann Pediatr Endocrinol Metab. 2022;28(3):178–83. 10.6065/apem.2244062.031 PMC10556445 35798297

[32] Burt Solorzano CM, McCartney CR. Obesity and the pubertal transition in girls and boys. Reproduction. 2010;140(3):399–410. 10.1530/REP-10-0119 PMC2931339 20802107

[33] Shah B, Tombeau Cost K, Fuller A, Birken CS, Anderson LN. Sex and gender differences in childhood obesity: contributing to the research agenda. BMJ Nutr Prev Health 2020;3(2):387–90. 10.1136/bmjnph-2020-000074 PMC7841817 33521549

[34] Lee D, Chung JM, Lee SD. Pediatric obesity and development of the penis and testis. Investig Clin Urol. 2024;65(2):189–95. 38454829 10.4111/icu.20230287 PMC10925733

[35] Ortega MT, McGrath JA, Carlson L, Flores Poccia V, Larson G, Douglas C et al. Longitudinal Investigation of Pubertal Milestones and Hormones as a Function of Body Fat in Girls. J Clin Endocrinol Metab. 2021;106(6):1668–83. 10.1210/clinem/dgab092 PMC8118584 33630047

[36] Lian Q, Mao Y, Luo S, Zhang S, Tu X, Zuo X, et al. Puberty timing associated with obesity and central obesity in Chinese Han girls. BMC Pediatr. 2019;19(1):1. 30606158 10.1186/s12887-018-1376-4 PMC6317212

[37] Chen C, Zhang Y, Sun W, Chen Y, Jiang Y, Song Y, et al. Investigating the relationship between precocious puberty and obesity: a cross-sectional study in Shanghai, China. BMJ Open. 2017;7(4):e014004. 28400459 10.1136/bmjopen-2016-014004 PMC5566589

[38] Li Y, Ma T, Ma Y, Gao D, Chen L, Chen M et al. Adiposity Status, Trajectories, and Earlier Puberty Onset: Results From a Longitudinal Cohort Study. J Clin Endocrinol Metab. 202;107(9):2462–72. 10.1210/clinem/dgac395 35779008

